# Clinical Application of Magnetic Resonance Spectroscopy in Diagnosis of Intracranial Mass Lesions

**DOI:** 10.1155/2021/6673585

**Published:** 2021-02-18

**Authors:** Callen Kwamboka Onyambu, Mufaddal Nuruddin Wajihi, Alfred Otieno Odhiambo

**Affiliations:** ^1^Department of Diagnostic Imaging and Radiation Medicine, University of Nairobi, Box 19676-00202, Nairobi, Kenya; ^2^Nairobi West Hospital, Box 15695-00509, Nairobi, Kenya

## Abstract

**Introduction:**

Conventional MR imaging provides highly detailed anatomic information with unrivalled soft tissue contrast making it the mainstay in the diagnosis of suspected brain lesions. Despite this, MRI alone at times cannot answer the diagnostic questions in quite a few patients. Proton MR Spectroscopy (H-MRS) provides information on the metabolic composition within an area under interrogation. By comparing the relative concentrations of specific metabolites, the neuroradiologist can deduce critical information regarding neuronal cell density and integrity, cell membrane turnover, metabolic fuel, and possible necrosis in the region of interest. This provides a biochemical picture of the underlying pathology and thus aids in the diagnosis.

**Methods:**

This was a cross-sectional comparative study.

**Results:**

Of the 63 patients examined by MRI and MRS for intracranial mass lesions, the radiologists were able to offer a single imaging diagnosis based on MRI alone in only 15 patients (23.8%) while when MRI imaging was combined with MR spectroscopy, a single imaging diagnosis was offered in 47 patients (74.6%). This was an overall statistically significant improvement.

**Conclusion:**

MRS aided the radiologist in offering a single diagnosis in high versus low-grade gliomas, high-grade gliomas versus tuberculomas, and recurrent tumours versus radiation necrosis.

## 1. Introduction

Conventional MR imaging provides a highly detailed anatomic depiction of the human body with unrivalled soft tissue contrast and has become the mainstay in the diagnosis of suspected brain lesions [1–3] Advances in MRI technology–including, but not limited to, Gadolinium enhanced imaging, diffusion weighted imaging, perfusion studies, and susceptibility weighted imaging–have improved the accuracy of MRI diagnoses considerably. In spite of these advancements and improved sensitivity, many brain lesions remain a diagnostic problem because of poor specificity [4]. Proton MR Spectroscopy (^1^H-MRS) provides complementary information, specifically the metabolic composition of the tissue under interrogation. While no less than 200 metabolites are generated in the brain, we are blinded to most. Proton MRS however employs mainly seven metabolites which are metabolically important and easily detectable. These principal metabolites and what they represent include lipids (Lip), lactate (Lac), N-acetyl aspartate (NAA), glutamine/GABA (Glx), creatine (Cr), choline (Cho), and myo-Inositol (mI).

By comparing the relative concentrations of these metabolites, the neuroradiologist can deduce critical information regarding neuronal cell density and integrity, cell membrane turnover, metabolic fuel, and possible necrosis in the region of interest, thereby, the likely underlying pathology [5]. The acquisition of MR spectroscopy only requires the relevant additional software and pulse sequences and an extended imaging time of only 10–12 minutes per patient. ^1^H-MR spectroscopy is a useful noninvasive add-on to MR imaging.

Using conventional MRI, an experienced neuroradiologist can accurately propose the likely histological diagnosis in 70–90% of brain lesions based on a variety of rather indirect imaging criteria and clinical data [1, 2]. These include age and clinical presentation, location, calcification, cyst formation, and contrast enhancement among others [6]. In the remaining 10–30%, the differentiation of ischaemic mass lesions, intra- and extra-axial brain tumours, discrimination between high- and low-grade tumours, and discrimination between neoplastic and nonneoplastic lesions, however, may be ambiguous if the diagnosis is exclusively based on these criteria [6].

In contrast to MRI, CT, and angiography, that is, methods that provide structural data, proton magnetic resonance spectroscopy (^1^H-MRS) provides nonanatomic information related to neuronal density and integrity, cell wall proliferation or degradation, energy metabolism [7], and necrotic transformation of brain and/or tumour tissues [8]. Hence, in conjunction with anatomic imaging modalities, ^1^H-MRS is playing an increasingly important role in the discrimination of a number of common neurological disorders such as stroke, epilepsy, multiple sclerosis, HIV, dementia, head injury, and near drowning [6].

Conventional 1.5 T MRI is available at the Kenyatta National Hospital (which is Kenya's main public referral hospital), and a few of the major private diagnostic imaging establishments in Nairobi and Mombasa. This is a significant improvement from only a few years back when there were only two low strength MRI scanners in the whole country and no public hospital had access to these services [9, 10]. Today, MRS services in Kenya are only available at three private establishments, only one of them offering multivoxel MRS. All other MRI providers, including the national hospital, do not provide MRS facilities.

## 2. Materials and Methods

All patients were investigated under a constant single-volume ^1^H-MRS protocol following structural MRI imaging. All imaging and spectroscopic studies were carried out with a 1.5 T Phillips Intera whole-body magnetic resonance scanner using a circularly polarized head coil.

Either T2 weighted or gadolinium enhanced T1 weighted axial images were used for voxel localization. Water-suppressed single-voxel spectra were acquired using a double spin-echo localization technique (PRESS, point-resolved excitation spin-echo sequence) with frequency-selective water suppression. ^1^H-MRS was performed using automated spectroscopy sequences at short TE of 31msec, and intermediate TE of 144msec. A voxel of 2–8 mL, depending on the size of the lesion, was placed within the lesion.

All MRI and MRS examinations were reported by the attending radiologists. PACS archived images and reports were used to record and analyse the imaging and spectroscopy findings.

### 2.1. Ethical Considerations

This study was done after obtaining approval from KNH-UON ethical review commission. Patient autonomy was maintained throughout the study.

## 3. Results

A total of 68 patients presented to Plaza Imaging Solutions for Brain MR Spectroscopy between September 2012 and September 2013. Of these, 63 patients satisfied the study's selection criteria and were thus recruited for the study.

The average age (mean (±SD)) of patients with intracranial masses was 45.33 years (±20.14). The youngest patient was 5 years of age while the oldest was 90 years old. Of the 63 patients, 32 (50.8%) were male and 31 (49.2%) were female, representing an almost equal 1 : 1 male to female ratio.

### 3.1. MRI Diagnosis

All patients referred for brain MRS underwent conventional brain MRI scans before their spectroscopy examinations. All MRI and MRS studies were reported and reviewed with a consultant specialist neuroradiologist who has more than 10-year practice experience in neuroradiology based on clinical information and imaging features. These MRI examinations were initially reported independently of their corresponding MRS findings. When the reporting radiologist had more than one differential, even if there were two indisputable differentials, this was recorded as “no single diagnosis.” The following diagnoses were made based on the clinical history provided and conventional MRI scans alone (Table 1).

It is clear that from all patients referred for brain MRS, a significant proportion (76.2%) (95% CI 63.8–86.0%) were unable to get a single definite diagnosis based on MRI alone.

### 3.2. Indication for MRS

**Figure 1:**
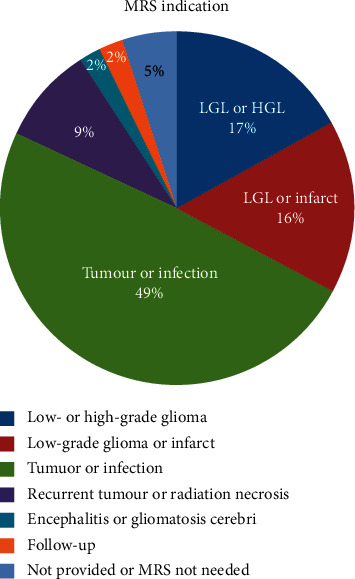
MRS indication.

Figure 1 lists the indications for which brain MR spectroscopies were performed during this study.

#### 3.2.1. Spectroscopy

The largest proportion of brain MR spectroscopies (49.2%) (*p*=0.899) were performed to help differentiate tumours from infections. Of these 31 indications, 30 (96.7%) (*p* < 0.001) were performed to differentiate tuberculoma, from glioblastoma multiforme (GBM). The remaining indication from this subgroup was to differentiate Primary CNS Lymphoma from toxoplasmosis in a patient with HIV infection. These statistics suggest that tuberculomas are statistically significantly the most common infectious intracranial mass lesions seen in this population. It is worth noting that no diagnosis of the pyogenic abscess was seen either based on MRI alone nor MRI + MRS during this study.

### 3.3. MRI + MRS Diagnosis

**Figure 2:**
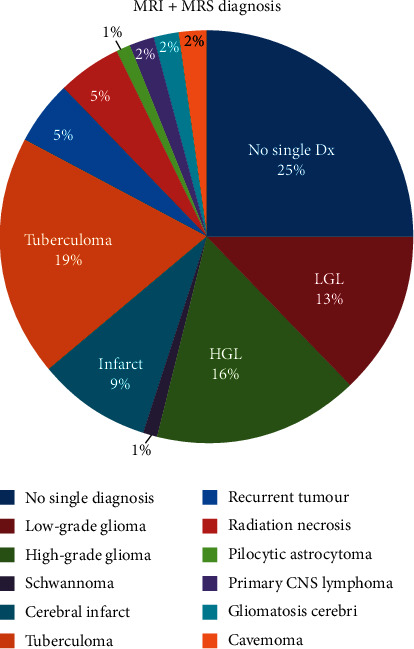
MRI + MRS diagnosis.

Figure 2 provides the diagnoses and their frequencies that were made after correlating the MRI findings to the findings of single-voxel MR spectroscopy performed on the intracranial mass lesions. It is important to note that only definitive imaging diagnoses were recorded as such. If there was more than one differential, this was recorded as “no single diagnosis.” These diagnoses were made by combining MRI and MRS findings, not from MRS alone. The two most frequent diagnoses made using MRI and MRS were high-grade gliomas (15.9%) (95% CI 7.9–27.3%) and tuberculomas (19%) (95% CI 10.3–30.9%).

#### 3.3.1. Efficacy of MRI + MRS

Of all patients examined by MRI and MRS for intracranial mass lesions, the radiologists were able to offer a single definite imaging diagnosis based on MRI alone in only 23.8% of patients while when MRI imaging was combined with MR spectroscopy, a single imaging diagnosis was offered in 74.6% of patients. This was an overall statistically significant improvement of 313.4% (*p* value <0.001), a slightly more than 3-fold improvement in diagnostic performance (Table 2). It is important to note that the majority of this population was biased towards patients who had been referred for MRS because of inconclusive prior imaging.

When we combine all individual diagnoses made using MRI alone and MRI + MRS, from all 63 patients, the addition of MRS improved the imaging diagnosis in 35 of these patients (55.6%). This means that the radiologist was able to rule out some differentials and offer a single imaging diagnosis or was, in a few cases, able to change the single diagnosis that was offered based on MRI alone. In 28 patients (44.4%), the addition of MRS was either not able to rule out other differentials or it did not change the single diagnosis that was offered based on MRI alone. Although this is an improvement, it is not statistically significant (*p*=0.374).

**Figure 3:**
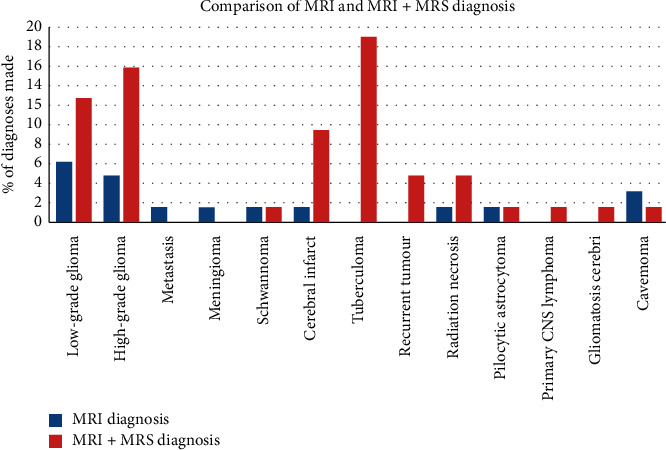
Comparison of MRI and MRI + MRS diagnosis.

The combination of MRI with MRS significantly increased the radiologist's chances of confidently diagnosing high-grade gliomas, low-grade gliomas, cerebral infarcts, tuberculomas, recurrent tumours, and radiation necrosis, rather than with MRI alone (*p* < 0.001 based on Wilcoxon Rank test) as demonstrated in Figure 3.

### 3.4. MRS Spectral Changes Based on Diagnosis

The metabolite changes are as follows.

Creatine was depressed in 70% of lesions diagnosed as high-grade glioma, while it was unchanged in all but one lesion diagnosed as low-grade glioma (*p*=0.025).

Decreased NAA, increased choline, lipid, and lactate were seen in both grades of tumours, with no significant difference in observed spectral changes. NAA was reduced and creatine was unchanged in both. Choline was increased in 75% of low-grade gliomas, while it was unchanged or decreased in 83.3% of cerebral infarcts. Although this trend is radiologically important for distinction between the two, the difference between the two groups was not statistically significant (*p*=0.149).

This is most likely due to the small frequency of these lesions, and a larger frequency would likely show a statistically significant difference. Lipid and lactate were increased in both. Myo-inositol was increased in 75% of lesions diagnosed as low-grade gliomas while, in cerebral infarcts, it was either increased or decreased or no change was seen.

Metabolite spectral changes between recurrent tumour and radiation necrosis showed no statistically significant differences in metabolite changes between the two groups likely due to the very small frequencies encountered of these lesions. However, from a radiological perspective, choline was increased in all (100%) of the recurrent tumours, while it was not increased in any diagnosis of radiation necrosis (*p*=0.071). Decreased NAA and increased lipid and lactate were seen in both groups.

**Figure 4:**
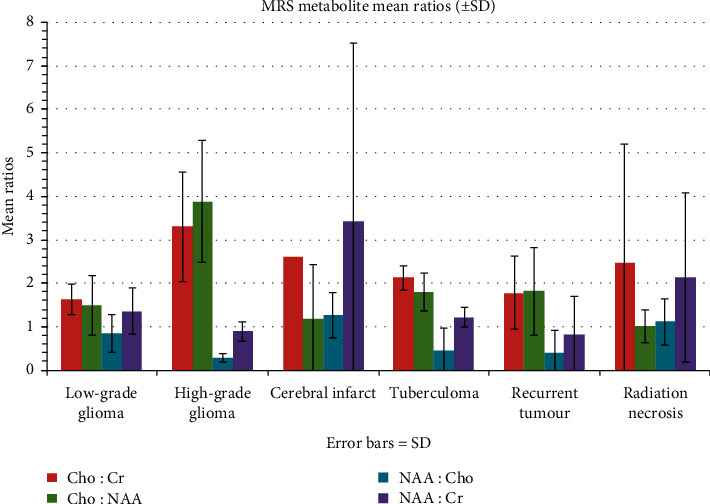
MRS ratios based on the diagnosis.

Comparison of metabolite spectral changes between high-grade gliomas and tuberculomas showing that the only statistically significant difference between the two groups was increased myo-inositol in 50% of high-grade glioma diagnoses, while, in all tuberculomas, myo-inositol was either unchanged or decreased (*p*=0.007). Decreased NAA, increased choline, lipid, and lactate were seen in both sets of diagnoses as shown in Figure 4.

Cho : Cr and Cho : NAA (at both TE 31 and 144 msec) were significantly higher in patients with high-grade glioma than in those with low-grade glioma. Conversely, NAA : Cho and NAA : Cr were significantly lower in patients with high-grade glioma than in those with low-grade glioma.

Similarly, patients with high-grade gliomas, tuberculomas, and recurrent tumours had significantly lower NAA : Cho ratios. NAA : Cr was increased in cerebral infarcts and radiation necrosis due to creatine depletion, while it was decreased in all other diagnoses. While NAA : Cr was decreased in both high-grade gliomas and tuberculomas, the decrease in high-grade gliomas was significantly more than in tuberculomas. Further analysis based on pairs of major diagnoses is provided below.

MRS was also useful in differentiating some tuberculomas from high-grade gliomas. Although increased choline was seen in both, Cho : Cr and Cho : NAA were significantly much higher in high-grade gliomas than in those confidently diagnosed as tuberculomas. NAA : Cr and NAA : Cho were reduced in both; however, only the reduction in NAA : Cr was statistically significantly more in high-grade gliomas than in tuberculomas (at TE 144 msec).

Brain MRS was also used to help differentiate low-grade gliomas from cerebral infarcts. The low frequency of cerebral infarcts diagnosed and the wide variation in ratios encountered meant that no statistically significant differences were seen between the two groups. However, NAA : Cr and NAA : Cho were much lower in low-grade gliomas compared to infarcts. The markedly reduced (almost zero) values of NAA and creatine, seen in several diagnosed cerebral infarcts (wide SD), caused deceptively elevated Cho : NAA (TE 31) and NAA : Cr and Cho : Cr ratios (TE 144).

A number of diagnoses of recurrent tumour versus radiation necrosis were made based on MRS findings. No statistically significant differences between the two groups were seen due to the low frequency of the occurrences. However, of note is the trend that NAA : Cr and NAA : Cho were much lower in recurrent tumour than in radiation necrosis, and Cho : NAA was much higher in recurrent tumour than in radiation necrosis. The very low levels of creatine in radiation necrosis caused deceptively elevated Cho : Cr ratios.

### 3.5. Selected MRI/MRS Images from Study Sample

**Figure 5:**
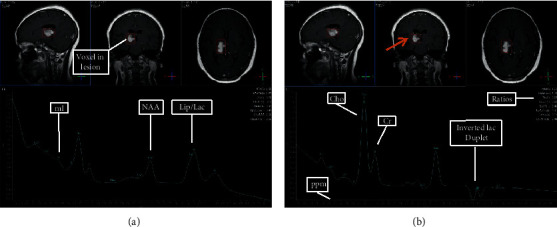
(a) TE 31 msec. (b) TE 144 msec. MRS was performed to differentiate between high-grade and low-grade glioma. The lesion was diagnosed as a high-grade glioma. NAA is decreased. Lipid and lactate are increased. Choline is markedly elevated. Cho : Cr ratio is 2.93, and Cho : NAA ratio is 2.44. At TE31, myo-inositol is reduced. Myo-inositol decrease has been associated with high-grade gliomas (58).

**Figure 6:**
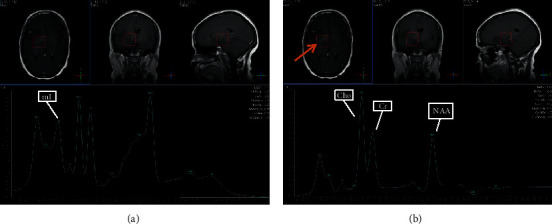
(a) TE 31 msec. (b) TE 144 msec. MRS was performed to differentiate between high-grade and low-grade glioma. The lesion was diagnosed as a low-grade glioma. NAA is depressed. Choline is moderately elevated. Cho : Cr is 1.67, and Cho : NAA is 1.90. Increased myo-inositol is also seen at TE31.

**Figure 7:**
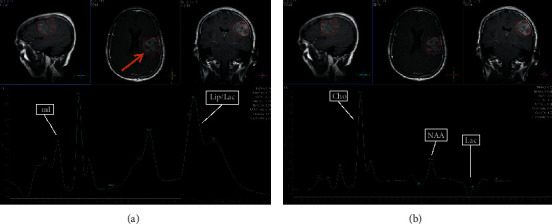
(a) TE 31 msec. (b) TE 144 msec. MRS was performed to differentiate between high-grade and low-grade glioma. The lesion was diagnosed as a high-grade glioma. NAA is reduced. Lipid and lactate are elevated. Choline is markedly elevated. Cho : Cr is 4.74, and Cho : NAA is 5.19. However, at TE31, myo-inositol is slightly increased. Myo-inositol increase has been associated with low-grade gliomas (58).

**Figure 8:**
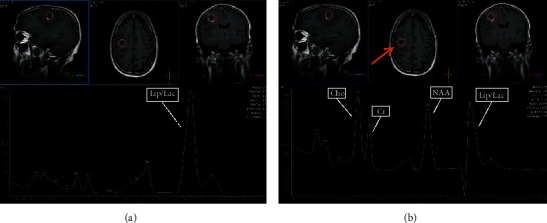
(a) TE 31 msec. (b) TE 144 msec. MRS was performed to differentiate between high-grade glioma and tuberculoma. The lesion was diagnosed as a tuberculoma. At TE31 prominent, lipid peak is seen at 1.3 ppm. At TE144, choline is increased. However, Cho : Cr is 1.57 and Cho : NAA is 1.20.

**Figure 9:**
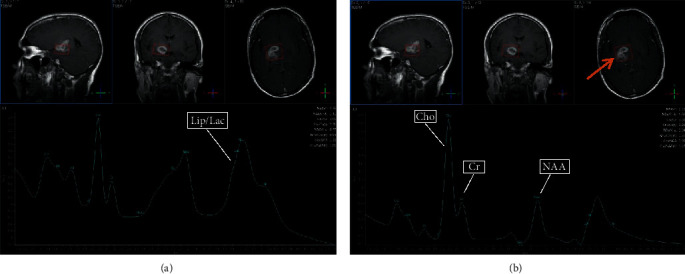
(a) TE 31 msec. (b) TE 144 msec. MRS was performed to differentiate between high-grade glioma and tuberculoma. The lesion was diagnosed as a high-grade glioma. NAA is depressed. Lipid is elevated. Choline is markedly elevated. Cho : Cr is 3.28, and Cho : NAA is 3.17. Note that in this case diagnosed as high0grade glioma, myo-inositol at TE31 is not decreased.

Images from selected cases representing the spectrum of findings encountered during the study are presented in Figures 5–9 below. All images shown are from actual cases of patients recruited in the study.

## 4. Discussion

Overall, the impact of combining MRI with MRS in improving the radiologist's ability to offer a single imaging diagnosis was significant. The number of “single diagnoses” increased by 3-fold (in 47/63) patients (74.6%) with MRI + MRS compared to MRI alone (15/63 patients (23.8%)). The most notable lesions, for which MRS aided the radiologist in offering a single diagnosis, were high and low-grade gliomas, tuberculomas, cerebral infarcts, recurrent tumours, and radiation necrosis. It is important to note that the majority of this sample population was biased towards patients who had been referred for MRS because of inconclusive prior imaging. This shows that, among the referring clinicians, there is awareness about the utility of and indications for brain MRS in the diagnosis of intracranial mass lesions.

Most patients presenting for brain MRS for intracranial mass lesions were in the age group of 41–50 years. By far the largest number of brain MR, spectroscopy examinations were performed to differentiate tuberculomas from high-grade gliomas, seeing that both may present as ring-enhancing mass lesions. We can infer that the age group of 41–50 years is like a watershed group which presents more of a challenge when differentiating tuberculomas from high-grade gliomas because epidemiologically high-grade gliomas tend to peak more in elderly patients, while tuberculomas tend to peak in younger patients [11–15].

Four major indications for brain MRS emerged from this study. These were to differentiate low- and high-grade gliomas; to differentiate tumours from infections (96.7% of these were to differentiate high-grade gliomas from tuberculomas); to differentiate low-grade gliomas from cerebral infarcts; and to differentiate recurrent tumour from radiation necrosis.

If we look at the efficacy of MRS added to MRI in improving the imaging diagnosis versus the four major indications described above, the addition of MRS to MRI did indeed improve the imaging diagnosis in all four indications. However, the improvement was not statistically significant (*p* > 0.05). It is possible that a larger sample size would probably show more statistically significant improvements.

When trying to differentiate between low-grade and high-grade gliomas, decreased NAA, increased Choline, lipid and lactate were seen in both grades of tumours, with no significant difference in observed spectral changes. Of statistical significance was the fact that creatine was depressed in a large proportion of diagnosed high-grade gliomas, while it remained unchanged in low-grade glioma diagnoses (*p*=0.025).

Of important note is that myo-inositol was increased in 87.5% of lesions diagnosed as low-grade gliomas. However, in lesions diagnosed as high-grade glioma, myo-inositol was decreased in only 30% of lesions, remained unchanged in 20%, and was increased in 50%. This contrasts the findings of Castillo et al. who reported a trend towards lower myo-inositol levels in high-grade gliomas compared to increased levels in those of low-grade gliomas [16].

More significant differences between low- and high-grade glioma diagnoses were seen when we look at metabolite ratios. Cho : Cr and Cho : NAA ratios were significantly higher in patients with high-grade glioma than in those with low-grade glioma. Conversely, NAA : Cho and NAA : Cr were significantly lower in patients with high-grade glioma than in those with low-grade glioma. Mean Cho : Cr and Cho : NAA ratios were <2.00 in low-grade gliomas [1.63 (±0.36)] and [1.48 (±0.68)] respectively, while they were >2.00 in high-grade gliomas [3.30 (±1.26)] and [3.88 (±1.40)], respectively. Mean NAA : Cr was <1.80 in low-grade gliomas [1.36 (±0.52)] and <1.00 in high-grade gliomas [0.89 (±0.23)], while mean NAA : Cho was <1.20 in low-grade gliomas [0.84 (±0.44)] and <0.50 in high-grade gliomas [±0.29 (0.10)]. Our findings compare favourably with those of Bertholdo et al. who report that at different institutions the threshold for Cho : Cr and Cho : NAA for differentiating low- and high-grade gliomas ranges between 2.0 and 2.5 [17].

The bulk of MRS examinations were performed to differentiate tuberculomas from high-grade gliomas. Based on MR spectrum appearance, decreased NAA, increased choline, lipid, and lactate were seen in both sets of diagnoses. Early work by Gupta et al. showed that tuberculomas tend to have prominent lipid peaks within the lesion, while all other metabolites are depressed [18]. However, our findings in tuberculomas compare to more recent work by Gupta et al. and Gutch et al. which show that Choline increase is also seen in tuberculomas depending on the degree of cellular infiltrate and caseation [19, 20].

The only statistically significant difference between the two groups was that myo-inositol was increased in 50% of high-grade glioma diagnoses, while, in all tuberculomas, myo-inositol was either unchanged or decreased (*p*=0.007).

Differentiating tuberculomas from high-grade gliomas can be particularly challenging based on the observed spectral changes of single-voxel MRS alone. Comparing metabolite ratios did assist in the differentiation. Although increased choline was seen in both, Cho : Cr and Cho : NAA were significantly much higher in high-grade gliomas, [3.30 (±1.26)] and [3.88 (±1.40)], respectively, than in those confidently diagnosed as tuberculomas [2.13 (±0.28)] and [1.80 (±0.43)]. NAA : Cr and NAA : Cho were reduced in both; however, only the reduction in NAA : Cr was statistically significantly more in high-grade gliomas [0.89 (±0.23)] than in tuberculomas [1.22 (±0.22)].

31(49.2%) of MR spectroscopies were performed to differentiate tumours from nontumours (infections). Of these, 30 (96.7%) were to differentiate tuberculomas from high-grade gliomas. The lowly diagnostic performance of MRS in this regard (51.6% improvement of imaging diagnosis) occurred mainly when elevated choline and lipids were seen in a lesion, and the Cho : NAA and Cho : Cr ratios were such that they could not be confidently diagnosed as one or the other. A large number (48.4%) of such MR spectroscopies led to “no single diagnosis,” with both differentials still being considered. The age of the patients was also a factor in this diagnostic dilemma as explained above. It is in these situations where multivoxel MRS if available would have been particularly useful. Multivoxel MRS would allow different parts of the lesion; the central area of breakdown, the enhancing solid rim, and the peritumoural region to be interrogated separately. Increased choline in the peritumoural oedema would greatly increase the confidence of diagnosing a high-grade glioma. Differentiation of tumour from nonneoplastic lesions is possible using NAA/Cho and NAA/Cr ratios, with high choline levels seen in the neoplastic lesions [21].

When multiple ring-enhancing lesions were encountered, this would favour tuberculomas; however, when such lesions had markedly elevated Choline and Cho : NAA and Cho : Cr ratios, it would be difficult to confidently diagnose tuberculomas, as a multifocal aggressive tumour like metastasis would also need to be considered in the differential.

Only one MRS was performed to differentiate between primary CNS lymphoma and toxoplasmosis in an HIV patient. No pyogenic abscess diagnosis was encountered.

The third indication for which MRSs were performed was to differentiate low-grade gliomas from cerebral infarcts, in those whose prior imaging was inconclusive. Choline was increased in 75% of low-grade gliomas, while it was unchanged or decreased in 83.3% of cerebral infarcts. Although this trend is radiologically important for distinction between the two, the difference between the two groups was not statistically significant (*p*=0.149). This is most likely due to the small frequency of these lesions encountered, and a larger frequency from a larger sample size would likely show a statistically significant difference. Another observation was that myo-inositol was increased in low-grade gliomas (75% of them), while in cerebral infarcts, no particular trend was seen although without any statistical significance.

MRS metabolite ratios showed no statistically significant differences between the two groups. However, mean NAA : Cr and NAA : Cho ratios were much lower in low-grade gliomas compared to infarcts. The markedly reduced (almost zero) values of NAA and creatine, seen in several diagnosed cerebral infarcts, caused deceptively elevated Cho : NAA (TE 31) and NAA : Cr and Cho : Cr ratios (TE 144). This suggests that the diagnosis of cerebral infarct versus low-grade glioma was more likely based on observed spectral changes of increased Choline in gliomas, rather than on metabolite ratios.

The indication for the fourth group of MRS examinations was to differentiate between recurrent tumour and radiation necrosis. Once again, due to the small frequencies encountered, no statistically significant differences in metabolite changes between the two groups were seen. However, from a radiological perspective, choline was increased in all (100%) of recurrent tumours, while it was not increased in any diagnosis of radiation necrosis (*p*=0.071).

However, of note is the trend that NAA : Cr and NAA : Cho were much lower in recurrent tumour than in radiation necrosis, and Cho : NAA was much higher in recurrent tumour than in radiation necrosis. The very low levels of creatine in radiation necrosis caused deceptively elevated Cho : Cr ratios.

One MRS examination was performed to help differentiate between encephalitis and gliomatosis cerebri which has a varied MRI appearance. MRS was useful in this instance as it showed typical MRS features of gliomatosis cerebri, which include elevated myo-inositol together with features of aggressive tumour, that is, increased choline, increased Cho : Cr and Cho : NAA ratios, and decreased NAA : Cr and NAA : Cho ratios [22].

One MRS examination was performed for a child whose MRI imaging diagnosis was a pilocytic astrocytoma. MRS findings only confirmed the MRI findings by showing typical accepted paradoxical findings of an aggressive-appearing metabolite pattern and increased choline and lactate, which do not reflect the histologically benign nature of this tumour [23]. MRS in this case did not however improve the imaging diagnosis.

The main study limitation is that findings were not correlated with histology. The correlation of MRS and relative cerebral blood volume (rCBV) has been shown to increase diagnostic accuracy to 100% when compared with histopathology [23].

## 5. Conclusions

MRS combined with MRI is an important tool in the arsenal of a radiologist for diagnosing intracranial mass lesions and provides a noninvasive way of interrogating the biochemical make-up of lesions within the brain. Overall, when patients are selectively chosen who have more than one differential based on conventional MRI, in whom MRS has the potential to answer the diagnostic question, MRS combined with MRI showed a threefold increase in the number of single imaging diagnosis offered by the radiologist.

The most notable lesions, for which MRS aided the radiologist in offering a single diagnosis, were high- and low-grade gliomas, tuberculomas, cerebral infarcts, recurrent tumours, and radiation necrosis.

MRS combined with MRI “improved” the imaging diagnosis in more than half of all patients examined. MRS is also valuable in differentiating between recurrent tumour and radiation necrosis based on observing increased spectra of choline in recurrent tumours, along with increased Cho : NAA ratios and decreased NAA : Cr and NAA : Cho ratios.

## 6. Limitations

One of the limitations of this study was the fact that we were unable to compare the imaging diagnosis with the histopathological diagnosis; hence, the investigator has referred to his findings as “imaging diagnosis” and not definitive diagnosis. As a result, we were unable to provide local data on the sensitivity and specificity of MRS for the diagnosis of intracranial mass lesions.

Secondly, while MRS findings showed trends in metabolite spectral changes and ratios for particular diagnoses and indication groups in one way or another, the fact that the frequencies of several diagnoses encountered were small, it was difficult to determine the statistical significance of these trends. A larger sample size with a longer study duration, or a study designed to target particular diagnoses, would be required to resolve this.

An important factor to consider is that majority of patients recruited in this study may already have had prior imaging with an inconclusive diagnosis. Due to cost implications, the decision to refer a patient for MRS is often made by the clinicians, either on their own or upon the recommendation of the radiologist. This, coupled with the fact that a consecutive series sample selection method was used, may have introduced an element of selection bias.

## Figures and Tables

**Table 1:** Diagnoses using MRI alone.

MRI diagnosis	Frequency (%)
No single diagnosis	48 (76.2)
Low-grade glioma	4 (6.3)
High-grade glioma	3 (4.8)
Metastasis	1 (1.6)
Meningioma	1 (1.6)
Schwannoma	1 (1.6)
Cerebral infarct	1 (1.6)
Radiation necrosis	1 (1.6)
Pilocytic astrocytoma	1 (1.6)
Cavernoma	2 (3.2)

**Table 2:** Overall diagnostic performance between MRI diagnosis and MRI + MRS diagnosis.

Findings	MRI diagnosis *n* (%)	MRI + MRS diagnosis *n* (%)	*P* value
Single imaging diagnosis	15 (23.8)	47 (74.6)	<0.001
No single imaging diagnosis	48 (76.2)	16 (25.4)	

## Data Availability

The supporting data are available in the University of Nairobi Repository and can be availed upon reasonable request. The data sets generated during this study are available from the corresponding author upon reasonable request.
